# Respiratory effects of electronic cigarette use in individuals who never smoked: A systematic review

**DOI:** 10.1016/j.clinme.2025.100295

**Published:** 2025-02-23

**Authors:** Grazia Caci, Arielle Selya, Giusy Rita Maria La Rosa, Lucia Spicuzza, Jaymin B. Morjaria, Giulio Geraci, Riccardo Polosa

**Affiliations:** a UOC MCAU, University Teaching Hospital “Policlinico-S.Marco”, University of Catania, Italy; b Tobacco Harm Reduction, Pinney Associates, Inc., Pittsburgh, PA, USA; c Center of Excellence for the Acceleration of HArm Reduction (CoEHAR), University of Catania, Catania, Italy; d Department of Clinical & Experimental Medicine, University of Catania, Catania, Italy; e Respiratory Unit - University Teaching Hospital “Policlinico-S.Marco”, University of Catania, Catania, Italy; f Department of Respiratory Medicine, Harefield Hospital, Guy's & St Thomas' NHS Foundation Trust, Harefield, UK; g Institute of Internal Medicine, University Teaching Hospital “Policlinico-S.Marco”, University of Catania, Catania, Italy; h Faculty of Medicine and Surgery, “Kore” University of Enna, Kore, Italy

**Keywords:** e-cigarettes, lung function, respiratory symptoms, systematic review

## Abstract

Current evidence on whether electronic cigarettes (ECs) pose respiratory risks is unclear, due to confounding by cigarette smoking; evidence among never-smoking individuals is needed. Following a narrative review and critical appraisal, a systematic review assessed possible respiratory outcomes prospectively associated with EC use among individuals who never smoked. Bias risk was evaluated using a Joanna Briggs Institute tool. Ten eligible studies examined outcomes of self-reported respiratory diagnosis, symptoms and lung function. Eight examined adults and three examined youth (with overlap). Overall, seven studies showed no significant association between respiratory outcomes and EC use among never-smoking individuals (*P*>0.05). Evidence for coughing and wheezing symptoms varied by model specification. Overall, EC use by never-smoking individuals is not associated with risk of severe respiratory outcomes, but may be associated with mild coughing/wheezing. Further research is needed using larger samples, long-term follow-ups (>5 years), and information on detailed patterns of EC use.

## Highlights

• Respiratory effects of e-cigarette (EC) use are confounded by smoking history.
• Evidence is needed among individuals who never smoked, to avoid confounding.
• A systematic review identified 10 eligible prospective studies.
• Exclusive EC use was not significantly associated with severe respiratory outcomes.
• However, there was a possible tenuous risk of coughing/wheezing.

## Graphical abstract

**Figure:**
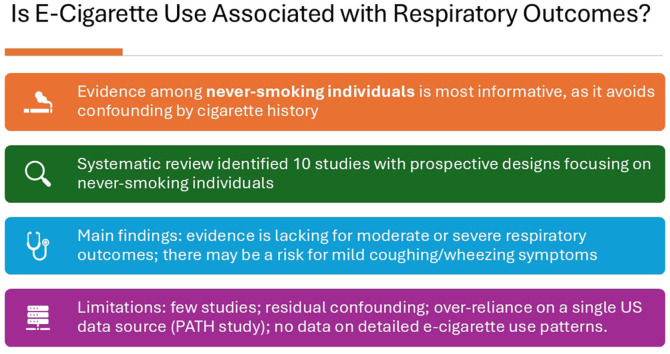

**Key practice implications**

Exclusive use of electronic cigarettes does not appear to be associated with severe respiratory risks, but may pose a risk of mild coughing and wheezing. E-cigarettes should be considered as a harm reduction tool among adults who smoke and are unlikely to quit.

Alt-text: Unlabelled box

## Introduction

Electronic cigarettes (ECs) are increasingly used for smoking cessation and harm reduction among adults who smoke,^1^, ^2^, ^3^ as they mimic the smoking experience without the production of harmful combustion or smoke.^4^^,^^5^ While ECs produce significantly lower exposures to harmful substances than tobacco cigarettes^6^, ^7^, ^8^ and are associated with lower nicotine dependence levels,^9^^,^^10^ concerns remain about dependence^11^ and health effects resulting from long-term EC use,^12^ especially by youth and tobacco-naïve individuals. EC toxicity likely varies by product-specific characteristics, including flavourings, though again, being non-combustible products, ECs are categorically less toxic.^12^^,^^13^

While data are not yet available for health outcomes requiring long-term cumulative exposures (eg ∼30 years for lung cancer), respiratory outcomes can plausibly develop over short- to medium-term durations (considered here as ∼1–5 years). Previous systematic and/or scoping reviews have reported that EC use is associated with lower toxic exposures than cigarettes^11^^,^^13^, ^14^, ^15^ but that there are some risks of respiratory irritation,^15^ mild adverse events^11^ and acute respiratory changes.^13^^,^^14^ However, none of these previous reviews robustly accounted for the confounding effect of prior cigarette smoking or focused specifically on individuals without an established smoking history. Further, the acute respiratory effects have unknown clinical significance, making the unique, clinical respiratory effects of ECs unclear from existing reviews.

Focusing on individuals without established smoking habits ('never-smoking'), therefore, is particularly informative for examining possible respiratory risks unique to EC use, as it avoids confounding by cigarette smoking history. However, EC use by never-smoking individuals is relatively rare.^16^, ^17^, ^18^, ^19^ Although the proportion of EC users who are smoking-naïve appears to be increasing as population-level nicotine consumption shifts from smoking to vaping, especially among younger age groups,^20^ this group nevertheless contributes very little to the evidence base at present. Accordingly, our group previously performed a narrative review and critical appraisal focusing on evidence among never-smoking adolescents and adults,^21^ concluding that there is some evidence of coughing or wheezing symptoms but that EC use is unlikely to pose significant or clinically meaningful respiratory harms over the medium term. However, these studies had important limitations, including an over-representation of US data (especially from the Population Assessment of Tobacco and Health (PATH)), inadequate controls for confounding (eg other combustible tobacco use), and limited follow-up durations.

Here, we extend this prior narrative review^21^ through a formal systematic review aimed at synthesising existing evidence on possible respiratory outcomes prospectively associated with EC use in never-smoking individuals. Due to the small number of qualifying studies, we considered all available respiratory outcomes (self-reported diagnoses, respiratory symptoms and lung function tests).

## Material and methods

### Seach strategy and selection criteria

This systematic review was reported following the Preferred Reported Items for Systematic Reviews and Meta-analyses (PRISMA) guidelines^22^ and the protocol was registered *a priori* in PROSPERO (CRD42024554721).

Our search used the following PICO (Population, Intervention, Comparator, Outcome) framework: P: Adults (≥18 years) and youth (12–17 years) with no established use of combustible cigarettes (ie never-smoking^1^); I: Current EC use; C: Non-current EC use (with former- vs. never-EC use combined or separate); O: Any self-reported or clinically-validated modification in respiratory function, including self-reported diagnosis (eg prevalence or incidence/onset) of respiratory disease (eg asthma); respiratory symptoms (prevalence, frequency or exacerbation), or lung function tests.

A comprehensive search in PubMed and Scopus was conducted in May 2024 using the keywords 'never smok*', 'näive', 'e-cig*', 'respir*' and 'asthma' (see Supplementary Table S1). The reference lists of included articles and review papers were also screened. Experts in tobacco harm reduction and/or respiratory function were consulted to confirm that all pertinent studies were included.

We included English-language studies with prospective designs only (clinical observational studies, randomized clinical trials (RCTs), and population surveys) to ensure the correct temporal sequence.

This review excluded studies that did not distinguish former-smoking from never-smoking, as lingering effects from prior smoking could confound results. We also excluded laboratory studies that measured acute exposures to EC aerosol, as these outcomes primarily document mild, transient respiratory symptoms (eg throat irritation, cough, wheezing, or modest changes in respiratory physiology and airway inflammation^23^, ^24^, ^25^) with unclear clinical relevance. Similarly, cellular and animal laboratory studies were excluded due to limited relevance to human clinical health^26^ and use of unrealistic operating conditions of EC devices which overstate negative impacts and lack appropriate experimental controls.^27^^,^^28^ Studies of heated tobacco products were excluded, as were reviews, study protocols, case reports and conference abstracts.

Two reviewers (GRMLR and GC) independently screened the titles and abstracts of all identified records to determine which studies required a full-text review; these were retrieved for further evaluation. The two reviewers and AS then independently assessed each full text to determine final eligibility. Any disagreements were resolved through discussion and consensus with an additional reviewer (RP).

### Data analysis

Data extraction and tabulation for each included study was carried out independently by two authors (GRMLR and AS), and the following information was tabulated: first author, year, country, study design, population, size (N) of current EC use group (ie among never-smoking individuals), size (N) of non-current EC use group (which may or may not distinguish former- and never-EC use), smoking behaviour verification, outcome, follow-up duration, main results, conclusions and funding.

A meta-analysis was not feasible due to heterogeneity in outcomes and the type of statistical estimate across studies (eg diagnoses vs. symptoms; prevalence vs. incidence) and the very small number of studies (<5) in each comparable group.

Risk of bias assessment was performed independently by two authors (GRMLR and GC) using the Joanna Briggs Institute's critical appraisal tools appropriate for the study design (*https://jbi.global/critical-appraisal-tools*). Any disagreement was resolved through consensus discussions or, if necessary, by consulting a third author (RP).

## Results

**Fig. 1:**
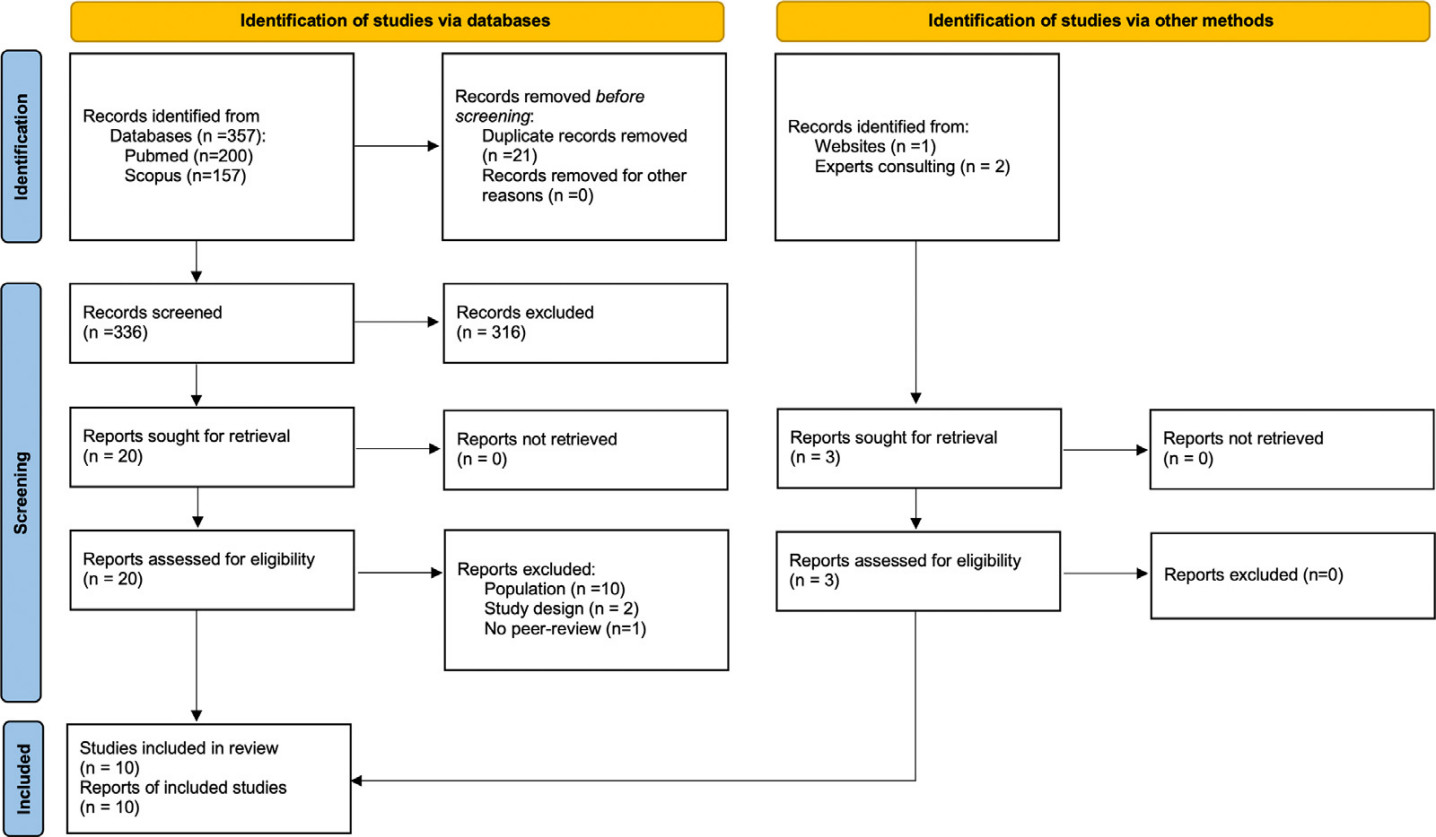
Search strategy.

Ten studies met inclusion criteria (Fig. 1). The list of excluded studies after full-text review is provided in Supplementary Table S2.

Table 1 presents the main characteristics of the included studies. Eight studies analysed adults (of which three focused only on younger adults, <24 or <30), and three studies analysed youth (one study examined both youth and adults). All studies defined EC use as current use (either 'some days' or 'every day' vs. 'not at all' or any past-30-day use). In six studies, the comparison group was non-current EC use (ie combining former- and never-EC use), while the remaining four studies distinguished former- and never-EC use. Most studies (n=8) analysed data from the US Population Assessment of Tobacco and Health (PATH) Study, one study analysed linked data between a Canadian population survey and administrative health records, and one study recruited a small cohort in Italy.

**Table 1:** General characteristics of the included studies.

First author, year, country study design	Population	Current e-cig use (never smokers) (N)	Non-current e-cig use (Former or/and never use among never smokers) (N)	Smoking behaviour verification	Outcome	Follow-up	Main results	Conclusions	Funding
Karey *et al*^23^ USA Nationally representative, longitudinal cohort survey (PATH)	Adults (≥18 years)	N=65 Any P30D e-cigarette use	Never e-cig use N=3,323 Former e-cig use N=250	Self-reported	Respiratory symptoms index (based on six wheezing items and a night-time dry cough item with higher values indicating more respiratory symptoms)	2 years Waves 4 (2016–2018) and 5 (2018–2019)	Reference: Never use AOR=1 Current e-cig use: AOR=0.82 95% CI [0.27–2.56] *P*=0.736	No significant association between e-cig use and important respiratory symptoms among never smokers was detected.	NR
Kenkel *et al*^29^ USA Nationally representative, longitudinal cohort survey (PATH) based on extension analysis of Bhatta and Glantz's^30^ results	Adults (aged ≥18 years)	N=12 Ever used an e-cigarette 'fairly regularly' and currently used e-cigarettes every day or some days	Never use N=2,705 Former use N=51	Self-reported	Lung or respiratory disease (self-reports of whether they had ever been told they have COPD, chronic bronchitis, emphysema or asthma)	1 year Waves 1 (2013–2014) and 3 (2015–2016)	Reference: Never use Coeff. LPM=−0.03 95% CI [−0.04, −0.01] The statistical software dropped data of the current e-cig users/never smokers from the model because the indicator for this category perfectly predicted the outcome – all 12 participants did not report incident respiratory diseases.^a^	Among never smokers, there was no evidence that current e-cig use was associated with respiratory disease.	Cornell University
Patel *et al*^31^ USA Nationally representative, longitudinal cohort survey (PATH)	Youths (aged 12–17 years)	N=142 F=54 M= 88 Any P30D e-cigarette use	Non-current use (N=8,590) F=4,346 M= 4,244	Self-reported	Asthma incidence (self-reported)	5 years Study Waves 1–5 (2013–2019)	Reference: Non-current use HR=1 Exclusive ENDS use Unadjusted HR=1.19 95% CI [0.73–1.96] *P*=0.477 Adjusted^a^ HR=1.25 95% CI [0.77–2.04] *P*=0.359	Short-term exclusive ENDS use was not statistically associated with higher risk of incident diagnosed asthma over 5 years.	National Cancer Institute (NCI) of the National Institutes of Health (NIH) and the Food and Drug Administration (FDA) Center for Tobacco Products.
Perez *et al* ^32^ USA Nationally representative, longitudinal cohort survey (PATH)	Adults (aged ≥18 years) and youths (aged 12–17 years)	Main analysis (based on first wave) Adults N=160 Youths N=96 Sensitivity analysis: Adults N=62 Youths N=59 Any P30D e-cigarette	No P30D ENDS use (never smokers at wave 1) Main analysis (based on first wave) Adults N=7,606 Youths N=16,927 Sensitivity analysis: Never use Adults N=5,355 Youths N=15,394	Self-reported	Asthma onset	Median (SE) Adults: 4.94 (0.06) years Youths: 4.19 (0.04) years Waves 1 (2013–2014) to 6 (2020–2021)	Main analysis: among participants reported never smoking at first wave: Reference: Non-current ENDS use Adults Crude association HR=9.57 95% CI [3.76–24.33] Model 3 HR=3.66 95% CI [1.23–10.85] Youths Crude association HR = 1.18 95% CI [0.46–2.70] Model 3 HR=1.55 95% CI [0.60–3.96] Sensitivity analysis: Reference: Non-current ENDS use and never-use of combustible tobacco at first wave HR=1 Current ENDS use at the first wave Adults Crude association HR=2.86 95% CI [0.36–22.82] Model 1 AHR=1.42 95% CI [0.14–14.59] Model 2 AHR=1.45 95% CI [0.14–15.04] Youths Crude association HR=0.66 95% CI [0.10–4.24] Model 1 AHR=0.70 95% CI [0.10–4.83] Model 2 AHR=0.68 95% CI [0.10–4.65]	Adults, but not youths, who reported never cigarettes and P30D ENDS use at the first wave showed higher risk of asthma incidence at earlier ages in comparison with those who reported no P30D ENDS use. There was no association between the P30D ENDS use with the age of asthma onset among adults and youths comparing users and never user (among never smokers at the first wave) in the sensitivity analysis.	National Heart, Lung, and Blood Institute and the US FDA Center for Tobacco Products.
Polosa *et al*^33^ Italy Prospective cohort study	Adults (≥18 years)	N=9 F=3 M=6 26.6±6.0 years Daily e-cigarette users of ≥3 months	Never e-cig use N=12 F=4 M=8 27.8±5.2 years	Self-reported	Lung function, respiratory symptoms, eNO, eCO and HRCT of the lungs	3.5 years	FEV1 (l, mean ± SD) Baseline; F/up 3 EC users: 3.82±0.78; 3.87±0.76 Control: 4.08±0.30; 4.11±0.30 *P*>0.05 FVC (l, mean±SD) Baseline; F/up 3 EC users: 4.93±0.95; 4.87±0.83 Control: 5.03±0.48; 5.02±0.42 *P*>0.05 FEV1/FVC (%, mean±SD) Baseline; F/up 3 EC users: 78.49±3.46; 79.08±2.83 Control: 81.45±5.03; 82.06±4.25 *P*>0.05 FEF25–75% (l/min, mean ± SD) Baseline; F/up 3 EC users: 3.29±0.70; 3.33±0.64 Control: 3.43±0.64; 3.56±0.58 *P*>0.05 eCO (ppm, median and IQ range) Baseline; F/up 3 EC users: 5.0 [3.5–7.3]; 4.0 [2.8–6.3] Control: 4.0 [3.5–7.5]; 5.0 [5.5–6.0] *P*>0.05 FeNO (ppb, median and IQ range) Baseline; F/up 3 EC users: 21.1 [16.2–24.5]; 20.0 [18.2–22.7] Control: 18.6 [17.6–25.7]; 20.0 [16.2–23.4] *P*>0.05 None of the participants referred any wheezing, shortness of breath, or chest tightness. Cough=2 EC user and 3 controls. No pathological findings were identified on HRCT of the lungs.	This small study showed no detectable modifications in lung health in never smokers who have been regularly vaping for at least 4 years.	University grant
Sanchez-Romero *et al*^34^ USA Nationally representative, longitudinal cohort survey (PATH)	Adults (≥18 years)	% (SE) Wave 1 (2013–2014) 0.3 (0.03) Wave 2 (2014–2015) 0.3 (0.03) Wave 3 (2015–2016) 0.3 (0.04) Wave 4 (2016–2018) 0.3 (0.03) Wave 5 (2018–2019) NA N=51^b^ (across waves 1–4) Currently used e-cigarettes on every day or some days	Noncurrent ENDS users % (SE) Wave 1 (2013–2014) 61.8 (0.62) Wave 2 (2014–2015) 58.6 (0.64) Wave 3 (2015–2016) 57.1 (0.66) Wave 4 (2016–2018) 56.3 (0.66) Wave 5 (2018–2019) NA N=∼9500–10,500^b^ (across waves 1–4)	Self-reported	Wheezing symptoms (self-reported)	5 years Waves 1 (September 2013 – December 2014) to 5 (December 2018 – November 2019)	Reference: Non-current use AOR=1 Current ENDS use: AOR=1.20 95% CI [0.83, 1.72] *P*=0.0.32 Supplementary analysis using 3-level ENDS use (never, former, current): Reference: Never use AOR=1 Current ENDS use: AOR=1.28 95% CI [0.89, 1.85] *P*=0.19	Exclusive ENDS use was not significantly associated with an increase in odds of self-reported wheezing compared with never use and non-current ENDS use among never smokers.	NCI of the NIH and FDA Center for Tobacco Products
Sargent *et al*^35^ USA Nationally representative, longitudinal cohort survey (PATH)	Young adults (18–24 years)	N=327 among former- and never-smokers (not separately reported) Any P30D e-cigarette use	N=5,888 Non-current e-cigarette use	Self-reported	Respiratory symptom index based on seven wheezing/cough questions from ISAAC with higher values indicating more respiratory symptoms. Cutoff values ≥2 and ≥3.	Waves 1 (2013–2014) to 2 (2014–2015) and 3 (2015–2016)	Reference: Never use Unadjusted Exclusive e-cig use RR=1.53 95% CI [0.98–2.40] Worsening symptoms (asymptomatic Wave 2- symptomatic Wave 3), adjusting for smoking history with never-smoking as the reference group: Cutoff≥2 RR=1.63 95% CI [1.02, 2.59] Cutoff≥3 RR=1.58 95% CI [0.84, 2.96] Improvement symptoms (symptomatic Wave 2- asymptomatic Wave 3), adjusting for smoking history with never-smoking as the reference group: Cutoff≥2 RR=0.57 95% CI [0.40, 0.82] Cutoff≥3 RR=1.64 95% CI [1.04, 2.58]	Compared to never users, exclusive users of e-cigs exhibited a similar risk of functionally important respiratory symptoms, independently from the cutoff considered. In exclusive e-cig use, worsening of symptoms depended from the symptoms severity and cutoff considered.	Federal funds from the National Institute on Drug Abuse, NIH, and the Center for Tobacco Products, FDA, Department of Health and Human Services
Stevens *et al*^36^ USA Nationally representative, longitudinal cohort survey (PATH)	Youths (aged 12–17 years)	N=54 (1.7%) Any P30D e-cigarette use	Never e-cig use N=2,998 (90.8%) Former e-cig use N=266 (7.5%)	Self-reported	Respiratory symptom index (based on responses for seven wheezing items with an index of ≥2 indicating functionally important respiratory symptoms)	1 year Waves 3 (October 2015 – October 2016) and 4 (December 2016 – January 2018)	Reference: Never use AOR=1 Former e-cig use: AOR=1. 20 95% CI [0.78, 1.85] *P*=0.411 Current e-cig use: AOR=0.86 95% CI [0.32, 2.32] *P*=0.767	E-cigarette use (including former and current) was not significantly associated with higher odds of a respiratory symptom at 1-year follow-up among never combustible tobacco users.	NCI at the NIH
To *et al*^37^ Canada Nationally representative, longitudinal cohort survey CCHS and health administrative databases (DAD and NACR)	Adults (aged 15–30 years)	N=75^b^ Any P30D e-cigarette use	N=365^a^^,^^b^ Non-current e-cigarette use	Self-reported	Asthma prevalence, asthma attacks (self-reported)	CCHS (cycles 2015–16 and 2017–18) and health administrative data (January 2015 – March 2018)	Reference: Non-current use Asthma prevalence: Current ENDS use, adjusting for smoking history with never-smoking as the reference group: AOR=1.21 95% CI [0.95, 1.54] *P*=0.1170	While there was no analysis specific to never-smokers, the adjusted model using never-smokers as the reference group found no association between current ENDS use and asthma prevalence or asthma attacks.^38^	The Canadian Institutes of Health Research Catalyst Grant: Health Effects of Vaping.
Xie *et al*^39^ USA Nationally representative, longitudinal cohort survey (PATH)	Young adults (18–24 years)	N=312 Ever used an e-cigarette 'fairly regularly' and currently used e-cigarettes every day or some days	N=8,388 Never used e-cigarettes N=1,140 Formerly used e-cigarettes	Self-reported	Respiratory symptoms (ie wheezing in the chest, and during or after exercise, dry cough at night)	1 year Waves 1 (2014–2015) to 5 (2018–2019) with exposure wave (waves 2–4) and outcome wave (waves 3–5)	Reference: Never use OR=1 Exclusive ENDS use Any respiratory symptom Fully adjusted OR=1.86 95% CI [1.35–2.58] Wheezing in the chest Fully adjusted OR=2.23 95% CI [1.28–3.91] Wheezing during exercise Fully adjusted OR=2.41 95% CI [1.39–4.16] Dry cough at night Fully adjusted OR=1.41 95% CI [0.97–2.04]	Among never smokers, current e-cig use was associated with 86% higher odds of reporting any respiratory symptom than never use. The association was particularly strong for wheezing in the chest and during exercise.	American Lung Association Public Policy Research Award, NHLBI grant and American Heart Association Tobacco Center for Regulatory Science grants

AOR, adjusted odds; CCHS, Canadian Community Health Survey; CI, confidence interval; DAD, Discharge Abstract Database; ENDS, electronic nicotine; eNO, exhaled breath nitric oxide, eCO, exhaled carbon monoxide, F, female; HR, Hazard Ratio; HRCT, high-resolution computed tomography; ISAAC, the International Study of Allergies and Asthma in Childhood; LPM, linear probability model; NACRS, National Ambulatory Care Reporting System; NCI, National Cancer Institute; NR, Not Reported; M, male; PATH, Population Assessment of Tobacco and Health; P30D, past 30-day.

a Adjusting for baseline age, sex, race/ethnicity, parental educational attainment, urbanicity, second-hand smoke exposure, household combustible tobacco use, and BMI-for-age.

b N values were estimated from other numbers in the paper, as they were not directly reported.

With respect to respiratory outcomes, four studies examined self-reported respiratory diagnosis, with two examining prevalence (one for any respiratory disease, one for asthma) and two examining incidence/onset of asthma (with one examining only *age* of onset). Six studies examined self-reported respiratory symptoms (most often wheezing; three used a threshold for functionally important symptoms). One study examined lung function outcomes based on spirometry tests and high-resolution computing tomography (HRCT).

As a meta-analysis was not feasible (see Methods), we qualitatively synthesise the findings, organising by type of outcome. First, of the four papers examining self-reported diagnosis, three focused on asthma specifically. To *et al*^37^ found no association between current (vs. non-current) EC use and *prevalence* of self-reported asthma (adjusted odds ratio (AOR)=1.21 [95% confidence interval (CI) [0.95–1.54]) among a sample of Canadian younger adults (ages 15–30) or past-year asthma attacks among those with asthma.^38^ There was an interaction with sex on past-year asthma *attacks*, such that female EC users (vs. male non-users) had higher odds of having an asthma attack (AOR=2.30[1.29–4.12]); however, there was a similar difference between female vs. male EC users (AOR=2.29[1.57–3.35]), so it is unclear whether this result is attributable to EC use or a sex difference. We also note that To *et al adjusted* for smoking status (using never-smoking as the reference group) rather than excluding formerly- and currently-smoking adults entirely; thus, results may not generalise to never-smoking adults specifically (see our narrative review^21^).

Patel *et al*^31^ and Perez *et al*^32^ both analysed outcomes of asthma incidence (ie new onset since baseline) using PATH data. Patel *et al*^31^ found that EC use among never-smoking youth (ages 12–17) was not significantly associated with onset of asthma 1 year later (adjusted hazard rate (AHR)=1.25[0.77–2.04]). Similarly, Perez *et al*^32^ did not find baseline EC use to be associated with age of asthma onset among youth naïve to smoking and asthma baseline (AHR=1.55[0.60–3.96]), though the association was significant among adults (age 18+) (AHR=3.66[1.23–10.85]). However, the latter became non-significant in a supplementary analysis that excluded participants with *any* baseline combustible tobacco product use (AHR=1.45[0.14–15.04]; see Discussion).

Finally, Kenkel *et al*^29^ performed a replication and extension of Bhatta & Glantz's PATH analysis^30^ of EC use and self-reported diagnoses of any respiratory disease (COPD, chronic bronchitis, emphysema or asthma). Unlike the original article, Kenkel *et al*^29^ differentiated between former- and never-smoking, and found no significant association between exclusive EC use and prevalence of respiratory disease over a 3-year period (though see Discussion for imprecision due to low numbers).

Together, these studies overall fail to support that exclusive EC use is associated with respiratory diagnoses among individuals who never smoked.

Six studies examined respiratory symptom outcomes. Polosa *et al*^33^ assessed respiratory symptoms (cough, wheezing, shortness of breath, and tight chest) prospectively over an average of 3.5 years among a small (N=21) cohort of adult EC users and non-EC-using controls; there was no evidence for respiratory symptoms from EC use alone (only one symptom, cough, was reported by two EC users and three controls; statistical test not performed due to small numbers). The remaining five studies all analysed PATH data. In never-smoking adults, Sanchez-Romero *et al*^34^ found that EC use at baseline was not significantly associated with higher odds of wheezing over 5 years (AOR=1.20[0.83–1.72]), and Karey et al.^40^ found no association with functionally important respiratory symptoms, using a threshold on a six-item index on wheezing and night-time dry cough (AOR=0.82[0.27–2.56]).

Sargent *et al*^35^ and Xie *et al*^39^ both examined self-reported respiratory symptoms in young adults (ages 18–24) in PATH. Sargent *et al*^35^ examined functionally important respiratory symptoms, defined using two different cut-offs (of 2+ and 3+ on a 0–9 scale) on a seven-item respiratory symptom index, as well as *worsening* and *improvement* of these symptoms over time. Baseline current EC use (vs. never-use) was not associated with different *likelihood* of functionally important respiratory symptoms but was, in some but not all models, significantly associated with *worsening* of symptoms (ie significant when using the 2+ cut-off (adjusted risk ratio (ARR)=1.63[1.02–2.59]), but not 3+ (ARR=1.58[0.84–2.96]). Findings on symptom *improvement* also varied in direction depending on the cut-off value (see Table 1). Like To *et al* ,^37^ Sargent *et al*^35^ estimates may be biased, as analyses *adjusted* for smoking status (using never-smoking as the reference group) rather than omitting former and current smoking groups entirely (see our narrative review^21^). In contrast, Xie *et al.*^39^ who use the same data source and age range above, report that current (vs. never) EC use at baseline was associated with higher odds of subsequent wheezing symptoms ('in the chest': AOR=2.23[1.28–3.91]; 'during exercise': AOR=2.41[1.39–4.16]) and any respiratory symptom (AOR=1.86[1.35–2.58]). However, these associations might reflect confounding by other tobacco use (see Discussion).

Stevens *et al*^36^ examined youth (ages 12–17) in PATH and outcomes of self-reported respiratory symptoms, using a seven-item index with a threshold of 2+ on a 0–9 scale, similar to Karey *et al*^40^ and Sargent *et al*^35^ above. Past 30-day EC use (vs. non-use) at baseline was not significantly associated important respiratory symptoms 1 year later among never-smoking youth (AOR=0.86[0.32–2.32]).

Overall, these six studies show somewhat mixed evidence: three studies (Karey *et al*^40^; Sanchez-Romero *et al*^34^ and Stevens *et al*^36^) reported a non-significant association with EC use; one (Polosa *et al*^33^) reported some coughing symptoms in both EC users and non-users, with unknown statistical significance; and two studies (Perez *et al*^32^ and Sargent *et al*^35^) reported tenuous associations whose statistical significance depended on model specifications.

Finally, Polosa *et al*^33^ was the only study to examine objective outcomes using lung function tests, biomarkers of airway inflammation (exhaled breath nitric oxide [eNO] and carbon monoxide [eCO]) and HRCT. In this prospective observational study of 31 never-smoking participants (16 daily EC users and 15 age- and sex-matched controls), there were no significant alterations in lung function or eNO. Furthermore, HRCT scans revealed no significant structural abnormalities in the lungs over an average observation period of 3.5 years.

The risk of bias for the included studies is reported in Supplementary Table S3. The observational study design prevents inferring causality, despite the longitudinal data.^29^, ^31^, ^32^^,^^34^, ^35^, ^36^, ^37^, ^39^, ^40^ Moreover, exposure and outcome data from the national surveys are self-reported, potentially introducing recall bias and reporting errors.^29^, ^31^, ^32^^,^^34^, ^35^, ^36^, ^37^, ^39^, ^40^ Although in general, studies reported analysis adjusting for some key confounders, other confounders were not assessed (eg allergies, flu, exposure to air pollution, family history of asthma, physical activity, or other environmental exposures). Additionally, the small sample sizes for never-smoking EC users limited the statistical power needed to detect potential associations. The follow-up time for outcome occurrence was judged 'unclear' in all studies, as sustained changes in respiratory function may take longer to develop and manifest. Finally, strategies to manage missing data were reported in only two studies.^32^^,^^35^

## Discussion

We performed a systematic review of 10 prospective studies on EC use and respiratory outcomes (including self-reported diagnoses, symptoms and lung function) among adolescents and adults who never smoked cigarettes. The majority analysed data from the US PATH study. Overall, *none* of the four studies examining self-reported respiratory diagnoses found a statistically significant association with baseline EC use among never-smoking individuals. Collectively, the six studies on respiratory symptoms did not show a significant association with EC use and severe respiratory symptoms; there was some tenuous evidence of an association with coughing and wheezing symptoms, though this was sensitive to model specifications (eg the exact cut-off of respiratory symptoms; how other combustible tobacco use was handled). One study on lung function tests showed no significant abnormalities or pathological findings among EC users who never smoked.^33^

Below, we discuss important considerations in interpreting this evidence. We refer readers to our more extensive prior narrative review and critical appraisal^21^ (which included many of the current eligible studies) for a detailed discussion of strengths and weaknesses, but summarise common themes here. First, knowledge of the timing of exposure and outcome is necessary to examine the possible causal relationship between EC use and respiratory disease. While prospective study can ensure the necessary temporal sequence, prospective study design alone is not sufficient, especially in the case of chronic respiratory conditions that may have preceded EC use. For example, asthma is often diagnosed in childhood.^41^ This limitation applies to To *et al*^37^ (as participants who already had asthma were included) and Sanchez-Romero *et al*^34^ (though this analysis reported a non-significant association regardless). Fortunately, several other studies employed a stronger design, by omitting participants who had pre-existing respiratory conditions at baseline that could have explained respiratory outcomes (Karey *et al*,^40^ Patel *et al*,^31^ Perez *et al,*^32^ Sargent *et al*,^35^ Stevens *et al*,^36^ Xie *et al*,^39^ and Reddy *et al*,^42^ which was not included in this review (see Supplementary Table S1 and our narrative review^21^).

Another important limitation is remaining statistical bias from other sources of confounding. In particular, most studies in this review did not account for use of other combustible tobacco product. An exception was a supplementary analysis by Perez *et al*:^32^ after further removing participants who used other tobacco products at baseline, there was no remaining association between exclusive EC use and asthma outcomes. Other unaccounted-for confounding factors (eg environmental pollutants) could further attenuate associations.

Two studies, To *et al*^37^ and Sargent *et al*,^35^ were retained here despite not restricting their analysis to never-smoking individuals, as the analysis allowed an estimation of EC's possible effect among never-smoking individuals (ie by adjusting for smoking status using never-smoking as the reference group); however, there may be remaining bias by the inclusion of formerly and currently smoking participants.^21^

Another limitation of existing evidence is small sample size due to the low prevalence of EC use among never-smoking individuals.^16^, ^17^, ^18^ For example, Polosa *et al*^33^, the one study identified in our review that examined objective tests of lung function, had a small sample size (n=9 never-smoking adults who used ECs). Similarly, Kenkel *et al*^29^ identified only 12 such cases – none of which had respiratory conditions at follow-up – preventing statistical analysis of this group. Thus, the absence of evidence in this review should not be interpreted as evidence for absence.

Other limitations of the studies reviewed include a limited follow-up period (1–5 years), over-reliance on US data (especially from PATH), and defining 'EC use' overly broadly rather than using detailed use patterns that would allow an assessment of cumulative, chronic use (see Selya *et al*^43^). Future research should prioritise longer follow-ups, independent samples (especially non-US samples), and collect more detailed EC use patterns that would be required to evaluate a dose–response relationship.

### Conclusions

This systematic review and qualitative synthesis indicate that EC use among never-smoking individuals may pose mild risks of coughing and wheezing, but evidence is lacking for more significant or clinically meaningful respiratory symptoms or harms over the short to medium term (∼1–5 years). Due to the limitations of available data, ongoing research on long-term and heavy EC use is warranted to monitor respiratory risks, especially research using larger and longer-term prospective studies,and assessing more detailed EC use patterns.

## Funding

The author(s) received no financial support for this article with the exception of the contribution from Department of Clinical and Experimental Medicine at the University of Catania to cover publication fees (UPB 6C725202048/2024).

## Declaration of competing interest

RP is full tenured professor of Internal Medicine at the University of Catania (Italy) and Medical Director of the Institute for Internal Medicine at the same University. He has received the following EU and governmental competitive grants: U-BIOPRED, AIR-PROM, Integral Rheumatology & Immunology Specialists Network (IRIS), Ministero dell’Università e della Ricerca (MUR) PNRR 3277/2021, PNRR 341/2022, and PNRR 411/2021 funded by NextGenerationEU of the European Commission. He has also received investigator-initiated grants from Foundation for a Smoke-Free World, Pfizer, GlaxoSmithKline, CV Therapeutics, NeuroSearch A/S, Sandoz, Merk Sharp & Dohme, Boehringer Ingelheim, Novartis, Arbi Group Srl., Duska Therapeutics, and Forest Laboratories. He is the founder of the Center for Tobacco Prevention and Treatment (CPCT) and of the Center of Excellence for the Acceleration of Harm Reduction at Catania University. He has received consultancy fees from Pfizer, Boehringer Ingelheim, Duska Therapeutics, Forest Laboratories, CV Therapeutics, Sermo Inc., GRG Health, Clarivate Analytics, Guidepoint Expert Network, and GLG Group. He receives textbooks royalties from Elsevier and EDRA. He is also Chair of the European Technical Committee for Standardization on “Requirements and test methods for emissions of electronic cigarettes” (CEN/TC 437; WG4) and scientific advisor of the non-profit Foundation RIDE2Med. Arielle Selya reports a relationship with Pinney Associates Pittsburgh that includes: employment, nonfinancial support, and travel reimbursement. Arielle Selya reports a relationship with Juul Labs Inc that includes: consulting or advisory and travel reimbursement. Arielle Selya reports a relationship with Phillip Morris International that includes: consulting or advisory. Arielle Selya reports a relationship with Global Action to End Smoking (formerly Foundation for a Smokefree World) that includes: consulting or advisory. If there are other authors, they declare that they have no known competing financial interests or personal relationships that could have appeared to influence the work reported in this paper. Jaymin Morjaria reports a relationship with Chiesi Pharmaceuticals Inc that includes: speaking and lecture fees. Jaymin Morjaria reports a relationship with Medtronic Minimally Invasive Therapies Mansfield that includes: non-financial support. Jaymin Morjaria reports a relationship with Pulmonx Corp that includes: non-financial support. If there are other authors, they declare that they have no known competing financial interests or personal relationships that could have appeared to influence the work reported in this paper. All other authors declare that they have no known competing financial interests or personal relationships that could have appeared to influence the work reported in this paper.

## CRediT authorship contribution statement

**Grazia Caci:** Writing – review & editing, Writing – original draft. **Arielle Selya:** Writing – review & editing, Writing – original draft, Methodology, Investigation, Data curation. **Giusy Rita Maria La Rosa:** Writing – review & editing, Validation, Methodology, Investigation, Data curation. **Lucia Spicuzza:** Writing – review & editing. **Jaymin B. Morjaria:** Writing – review & editing. **Giulio Geraci:** Writing – review & editing, Writing – original draft. **Riccardo Polosa:** Writing – review & editing, Writing – original draft, Supervision, Conceptualization.

## Appendix Supplementary materials

Image, application 1

Image, application 2

Image, application 3

## Footnotes

1 “Never-smoking” as used here includes both never-*established* smoking (i.e., <10+ cigarettes/lifetime, as typically assessed in adults) and true never-smoking (i.e., not even a puff; typically assessed in youth).
