# Supplementary material for: Respiratory effects of electronic cigarette use in individuals who never smoked: A systematic review
Source: Clin Med (Lond). 2025 Feb 23;25(2):100295. doi: 10.1016/j.clinme.2025.100295 (PMC11930579; doi:10.1016/j.clinme.2025.100295)
Supplement: Supplementary file 1 [file mmc1.docx]

**Supplementary Table S2.** List of excluded studies after review of full text

| **STUDY** | **REASON FOR EXCLUSION** |
| --- | --- |
| 1. Zavala-Arciniega L, Cook S, Hirschtick J, Xie Y, Mukerjee R, Arenberg D, Barnes GD, Levy DT, Meza R, Fleischer N. Longitudinal associations between exclusive, dual and polytobacco use and respiratory illness among youth. Res Sq [Preprint]. 2024 Jan 22:rs.3.rs-3793149. doi: 10.21203/rs.3.rs-3793149/v1. | Not peer-reviewed |
| 1. Delmas MC, Pasquereau A, Renuy A, Bénézet L, Ribet C, Zins M, Guignard R, Pérez T, Roche N, Leynaert B. Electronic cigarette use and respiratory symptoms in the French population-based Constances cohort. Respir Med. 2024 Jan;221:107496. doi: 10.1016/j.rmed.2023.107496. | Study design (no longitudinal data) |
| 1. Mukerjee R, Hirschtick JL, Arciniega LZ, Xie Y, Barnes GD, Arenberg DA, Levy DT, Meza R, Fleischer NL, Cook SF. ENDS, Cigarettes, and Respiratory Illness: Longitudinal Associations Among U.S. Youth. Am J Prev Med. 2024 May;66(5):789-796. doi: 10.1016/j.amepre.2023.12.005. | Population (no never smokers) |
| 1. Cheney MK, Song H, Bhochhibhoya S, Lu Y. Chronic disease as a risk factor for cigarette and e-cigarette use from young adulthood to adulthood. Prev Med Rep. 2023 Oct 14;36:102473. doi: 10.1016/j.pmedr.2023.102473. | Population (no never smokers) |
| 1. Tackett AP, Urman R, Barrington-Trimis J, Liu F, Hong H, Pentz MA, Islam TS, Eckel SP, Rebuli M, Leventhal A, Samet JM, Berhane K, McConnell R. Prospective study of e-cigarette use and respiratory symptoms in adolescents and young adults. Thorax. 2024 Jan 18;79(2):163-168. doi: 10.1136/thorax-2022-218670. | Population (no never smokers) |
| 1. Mattingly DT, Cook S, Hirschtick JL, Patel A, Arenberg DA, Barnes GD, Levy DT, Meza R, Fleischer NL. Longitudinal associations between exclusive, dual, and polytobacco use and asthma among US youth. Prev Med. 2023 Jun;171:107512. doi: 10.1016/j.ypmed.2023.107512. | Population (no never smokers) |
| 1. Chaiton M, Pienkowski M, Musani I, Bondy SJ, Cohen JE, Dubray J, Eissenberg T, Kaufman P, Stanbrook M, Schwartz R. Smoking, e-cigarettes and the effect on respiratory symptoms among a population sample of youth: Retrospective cohort study. Tob Induc Dis. 2023 Jan 21;21:08. doi: 10.18332/tid/156839. | Study design (no longitudinal data) |
| 1. Berlowitz JB, Xie W, Harlow AF, Blaha MJ, Bhatnagar A, Benjamin EJ, Stokes AC. Cigarette‒E-cigarette Transitions and Respiratory Symptom Development. Am J Prev Med. 2023 Apr;64(4):556-560. doi: 10.1016/j.amepre.2022.10.006. | Population (no never smokers) |
| 1. Cordova J, Pfeiffer RM, Choi K, Grana Mayne R, Baker L, Bachand J, Constantine K, Altekruse S, Reyes-Guzman C. Tobacco use profiles by respiratory disorder status for adults in the wave 1-wave 4 population assessment of tobacco and health (PATH) study. Prev Med Rep. 2022 Oct 12;30:102016. doi: 10.1016/j.pmedr.2022.102016. | Population (no never smokers) |
| 1. Dai H, Khan AS. A Longitudinal Study of Exposure to Tobacco-Related Toxicants and Subsequent Respiratory Symptoms Among U.S. Adults with Varying E-cigarette Use Status. Nicotine Tob Res. 2020 Dec 15;22(Suppl 1):S61-S69. doi: 10.1093/ntr/ntaa180. | Population (no never smokers) |
| 1. Tackett AP, Keller-Hamilton B, Smith CE, Hébert ET, Metcalf JP, Queimado L, Stevens EM, Wallace SW, McQuaid EL, Wagener TL. Evaluation of Respiratory Symptoms Among Youth e-Cigarette Users. JAMA Netw Open. 2020 Oct 1;3(10):e2020671. doi: 10.1001/jamanetworkopen.2020.20671. | Population (no never smokers) |
| 1. Bhatta DN, Glantz SA. Association of E-Cigarette Use With Respiratory Disease Among Adults: A Longitudinal Analysis. Am J Prev Med. 2020 Feb;58(2):182-190. doi: 10.1016/j.amepre.2019.07.028. | Population (no never smokers) |
| 1. Reddy KP, Schwamm E, Kalkhoran S, Noubary F, Walensky RP, Rigotti NA. Respiratory Symptom Incidence among People Using Electronic Cigarettes, Combustible Tobacco, or Both. Am J Respir Crit Care Med. 2021 Jul 15;204(2):231-234. doi: 10.1164/rccm.202012-4441LE. | Population (no never smokers) |
