# Supplementary material for: Respiratory effects of electronic cigarette use in individuals who never smoked: A systematic review
Source: Clin Med (Lond). 2025 Feb 23;25(2):100295. doi: 10.1016/j.clinme.2025.100295 (PMC11930579; doi:10.1016/j.clinme.2025.100295)
Supplement: Supplementary file 2 [file mmc2.docx]

**Supplementary Table S3.** Risk of bias assessment of the included studies

|  | **JBI CRITICAL APPRAISAL CHECKLIST FOR COHORT STUDIES** | | | | | | | | | | |
| --- | --- | --- | --- | --- | --- | --- | --- | --- | --- | --- | --- |
| **Study** | **1** | **2** | **3** | **4** | **5** | **6** | **7** | **8** | **9** | **10** | **11** |
| Karey et al. 2024 | Y | Y | N | N | N | U | N | U | N | N | Y |
| Kenkel et al. 2020 | Y | Y | N | N | N | U | N | U | N | N | Y |
| Patel et al. 2023 | Y | Y | N | N | N | U | N | U | N | N | Y |
| Perez et al. 2023 | Y | Y | N | N | N | U | N | U | N | Y | Y |
| Polosa et al. 2017 | Y | Y | N | N | N | Y | Y | U | N | N | Y |
| Sanchez-Romero et al. 2023 | Y | Y | N | N | N | U | N | U | N | N | Y |
| Sargent et al. 2022 | Y | Y | N | N | Y | U | N | U | N | Y | Y |
| Stevens et al. 2022 | Y | Y | N | N | N | U | N | U | Y | NA | Y |
| To et al. 2023 | Y | Y | N | N | N | U | N | U | U | U | Y |
| Xie et al. 2022 | Y | Y | N | N | Y | U | N | U | N | N | Y |

*Legend*

*1. Were the two groups similar and recruited from the same population?*

*2. Were the exposures measured similarly to assign people to both exposed and unexposed groups?*

*3. Was the exposure measured in a valid and reliable way?*

*4. Were confounding factors identified?*

*5. Were strategies to deal with confounding factors stated?*

*6. Were the groups/participants free of the outcome at the start of the study (or at the moment of exposure)?*

*7. Were the outcomes measured in a valid and reliable way?*

*8. Was the follow up time reported and sufficient to be long enough for outcomes to occur?*

*9. Was follow up complete, and if not, were the reasons to loss to follow up described and explored?*

*10. Were strategies to address incomplete follow up utilized?*

*11. Was appropriate statistical analysis used?*

*Y: Yes; N: No; U: Unclear; NA: Not Applicable*
