# Supplementary material for: Respiratory effects of electronic cigarette use in individuals who never smoked: A systematic review
Source: Clin Med (Lond). 2025 Feb 23;25(2):100295. doi: 10.1016/j.clinme.2025.100295 (PMC11930579; doi:10.1016/j.clinme.2025.100295)
Supplement: Supplementary file 3 [file mmc3.docx]

**Table S1.** Search strategy for each database

| **Database** | **Strategy** | **Date** |
| --- | --- | --- |
| **Pubmed** | ("never-smokers"[All Fields] OR "never smokers"[All Fields] OR "never smok*"[All Fields] OR "naive"[All Fields] OR "adults"[All Fields] OR "healthy"[All Fields] OR "youth*"[All Fields] OR "young"[All Fields] OR "never used cigarettes"[All Fields] OR "never smoked"[All Fields]) AND ("electronic nicotine delivery systems"[MeSH Terms] OR "electronic nicotine delivery systems"[All Fields] OR e-cigarette[All Fields] OR "e cig*"[All Fields] OR vaping [MeSH Terms] OR vaping[All Fields] OR "ENDS"[All Fields] OR "electronic cigarette*"[All Fields]) AND ("respir*"[All Fields] OR “lung” [MeSH Terms] OR "lung"[All Fields] OR "asthma"[MeSH Terms] OR "asthma"[All Fields]) AND ("cohort"[All Fields] OR "observational"[All Fields] OR "longitudinal"[All Fields] OR "follow-up"[All Fields] OR "randomized controlled trial"[Publication Type] OR "randomized controlled trials as topic"[MeSH Terms] OR "randomized controlled trial"[All Fields] OR "RCT"[All Fields]) | 27/05/2024 |
| **Scopus** | TITLE-ABS-KEY ( ( "never-smokers" OR "never smokers" OR "never smok*" OR "never used cigarettes" OR "never smoked" ) AND ( "e-cig*" OR "e-cigarette" OR ends OR "electronic nicotine delivery systems" OR "electronic cigarette*" ) AND ( "respir*" OR "lung" OR "asthma" ) AND ( "cohort" OR "observational" OR "longitudinal" OR "follow-up" OR "randomized controlled trial" OR "RCT" ) ) | 27/05/2024 |
